# A Two-Dimensional Computer-Aided Design Study of Unclamped Inductive Switching in an Improved 4H-SiC VDMOSFET

**DOI:** 10.3390/mi15010035

**Published:** 2023-12-23

**Authors:** Xinfeng Nie, Ying Wang, Chenghao Yu, Xinxing Fei, Jianqun Yang, Xingji Li

**Affiliations:** 1 Key Laboratory of RF Circuits and Systems, Ministry of Education, Hangzhou Dianzi University, Hangzhou 310018, China; 13672121151@163.com (X.N.);; 2 Yangzhou Marine Electronic Instrument Institute, Yangzhou 225001, China; 3 National Key Laboratory of Materials Behavior and Evaluation Technology in Space Environment, Harbin Institute of Technology, Harbin 150080, China

**Keywords:** 2D numerical simulation, SiC, avalanche, UIS, power MOSFET

## Abstract

Due to its high thermal conductivity, high critical breakdown electric field, and high power, the silicon carbide (SiC) metal-oxide-semiconductor field-effect transistor (MOSFET) has been generally used in industry. In industrial applications, a common reliability problem in SiC MOSFET is avalanche failure. For applications in an avalanche environment, an improved, vertical, double-diffused MOSFET (VDMOSFET) device has been proposed. In this article, an unclamped inductive switching (UIS) test circuit has been built using the Mixed-Mode simulator in the TCAD simulation software, and the simulation results for UIS are introduced for a proposed SiC-power VDMOSFET by using Sentaurus TCAD simulation software. The simulation results imply that the improved VDMOSFET has realized a better UIS performance compared with the conventional VDMOSFET with a buffer layer (B-VDMOSFET) in the same conditions. Meanwhile, at room temperature, the modified VDMOSFET has a smaller on-resistance (R_on,sp_) than B-VDMOSFET. This study can provide a reference for SiC VDMOSFET in scenarios which have high avalanche reliability requirements.

## 1. Introduction

Some important progress towards wide-bandgap semiconductors has been described in [1,2,3]. Silicon carbide (SiC) metal-oxide-semiconductor field-effect transistors (MOSFET) have been widely used in large quantities due to their excellent properties, such as, high thermal conductivity, high critical breakdown electric field, and high power [4,5,6]. In the application of SiC MOSFET, a common reliability problem is avalanche failure.

The standard test commonly used to evaluate the avalanche robustness of SiC MOSFET is the unclamped inductive switching (UIS) test [7]. So far, in order to ensure the reliability of SiC MOSFET avalanche failure, all kinds of studies have been conducted to assess the avalanche robustness of SiC MOSFET. In [8,9,10,11], the researchers show that UIS failure appears when the temperature of the source aluminum layer surpasses the melting point. In addition, the switching on of parasitic bipolar junction transistor (BJT) is also thought to be a cause of avalanche failure in [12,13]. And in [14], the researchers believe that the opening of the channel is also responsible for the avalanche failure. 

Moreover, Bai et al. [15] studied the effects of structural parameters on the characteristics of UIS. The researchers discussed the influence of temperature on avalanche-energy-processing capacity and established a safe avalanche operating boundary model in [16,17]. Some researchers have studied the failure mechanism and avalanche ability of different device structures under single-pulse UIS stress, such as planar gate, asymmetric, and double-trench SiC MOSFET [18,19,20,21]. There is also some research on the failure mechanism and performance degradation of SiC MOSFET under repetitive avalanche-shock conditions [22,23,24,25,26,27,28]. In addition, for the purpose of improving the avalanche robustness of SiC MOSFET, some modified designs were proposed by the authors of [29].

In this paper, a concept for a device structure which can improve avalanche capability is proposed. A buffer layer is added to adjust the high electric field in the N-drift [30]. Next, we ensured a decrease in ON resistance by adding the current spread layer (CSL). Then, we added a high concentration P-type region and low concentration N-type regions to the device to suppress the triggering of parasitic BJT. The avalanche energy in the proposed structure can be increased by 20% compared with the traditional structure, and the R_on,sp_ is reduced to 1.0 mΩ·cm^2^ by adding the CSL layer.

## 2. Description of Device Structure and Simulation

**Figure 1:**
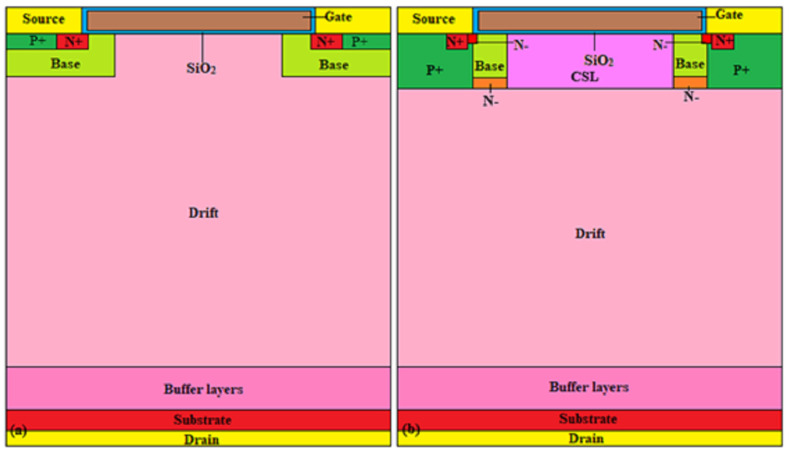
Cross-sectional view of 1.7-kV SiC MOSFET (**a**) B-VDMOSFET, (**b**) DPNB-VDMOSFET.

A cross-sectional view of the 4H-SiC VDMOSFET with a buffer layer (B-VDMOSFET) is shown in Figure 1a. A cross-sectional scheme of the improved VDMOSFET with CSL, deep p+ region, n− regions, and a buffer layer (DPNB-VDMOSFET) is shown in Figure 1b.

The doping concentration of the N-drift is 3 × 10^15^ cm^−3^. The P-base region is at a constant impurity concentration of 2 × 10^17^ cm^−3^. The impurity concentration in the substrate is 1 × 10^19^ cm^−3^. And the area factor is 4 × 10^5^.

The design of the 1.7-kV basic SiC VDMOSFET parameter refers to [31,32]. The main parameters are shown in Table 1.

**Figure 2:**
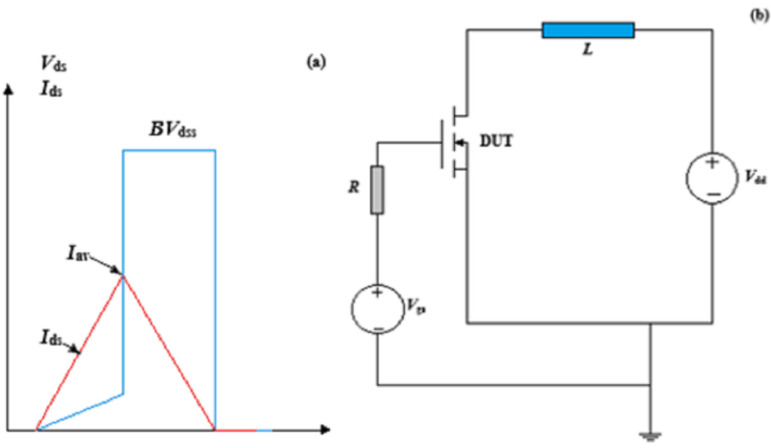
(**a**) Test waveform; (**b**) test circuit.

In addition, the UIS test circuit has been built using the Mixed-Mode simulator in TCAD simulation software. Figure 2a,b show both the test circuit and the test waveform.

## 3. Numerical Simulation Results and Discussion

### 3.1. Basic Electrical Characteristics

**Figure 3:**
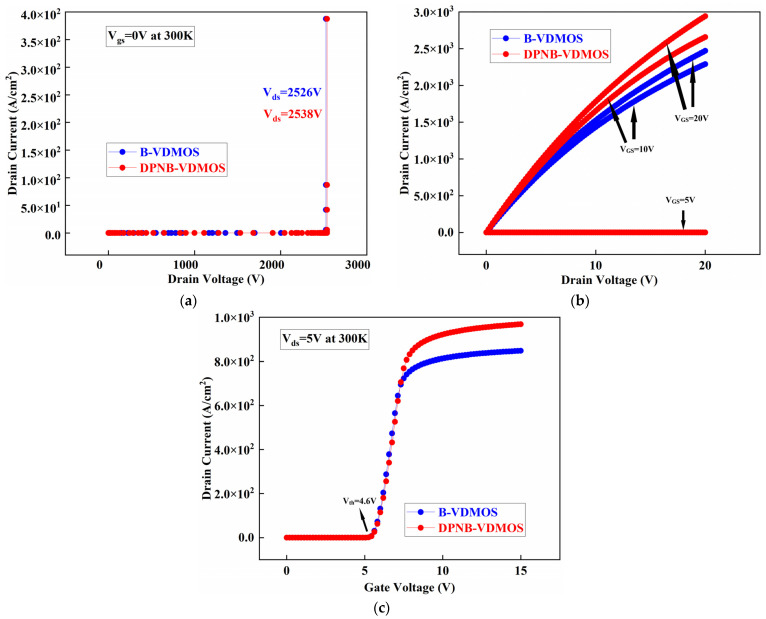
(**a**) The breakdown voltage characteristics of devices (**b**) ON-state characteristic curves for the devices. (**c**) Transfer characteristic curves for the devices.

Figure 3a shows the breakdown voltage characteristics of two MOSFETs. The breakdown voltages in the B-VDMOSFET and DPNB-VDMOSFET are 2526 V and 2538 V. Figure 3b shows the ON-state performance of the B-VDMOSFET and DPNB-VDMOSFET. However, the modified structure has better conduction characteristics due to the current path in the CSL region. The R_on,sp_ of the B-VDMOSFET and DPNB-VDMOSFET are 5.8 mΩ·cm^2^ and 4.8 mΩ·cm^2^ at V_GS_ = 20 V. As shown in Figure 3c, because their base regions have an equal doping concentration, the threshold voltages (V_th_) of two SiC MOSFETs are identical.

### 3.2. UIS Characteristic

Analyses of the UIS performance of the two SiC VDMOSFET were carried out using the 2D simulator software Sentaurus TCAD. These simulation experiments were carried out using specific physical models and material properties of SiC. Some of the pivotal physical models include: (1) the Fermi model is used to describe the carrier distribution of two devices. (2) the Shockley Read Hall (SRH) and Auger recombination models are concerned with temperature and doping. (3) The incomplete ionization model is used to calculate the impact ionization rate. (4) The Analytic model is used to describe the temperature dependent mobility of carrier distributions [33,34,35,36].

## 4. UIS Simulation Results and Discussion

In an SiC-power MOSFET, the SiC material does not melt but sublimes at temperatures higher than 2200–2300 K, the metal/SiC surface contact or oxide could be damaged by local overheating at the temperatures above 1500 K.

In order to compare the avalanche ability of the B-VDMOSFET and DPNB-VDMOSFET, UIS simulation experiments were carried out at room temperature with 10 mH of load inductance, 20 Ω of resistance and a V_ds_ of 80 V. The last waveform before avalanche failure is usually used to calculate the avalanche tolerance E_av_ of a device. Avalanche tolerance E_av_ is an important parameter used to evaluate the reliability of device UIS.

**Figure 4:**
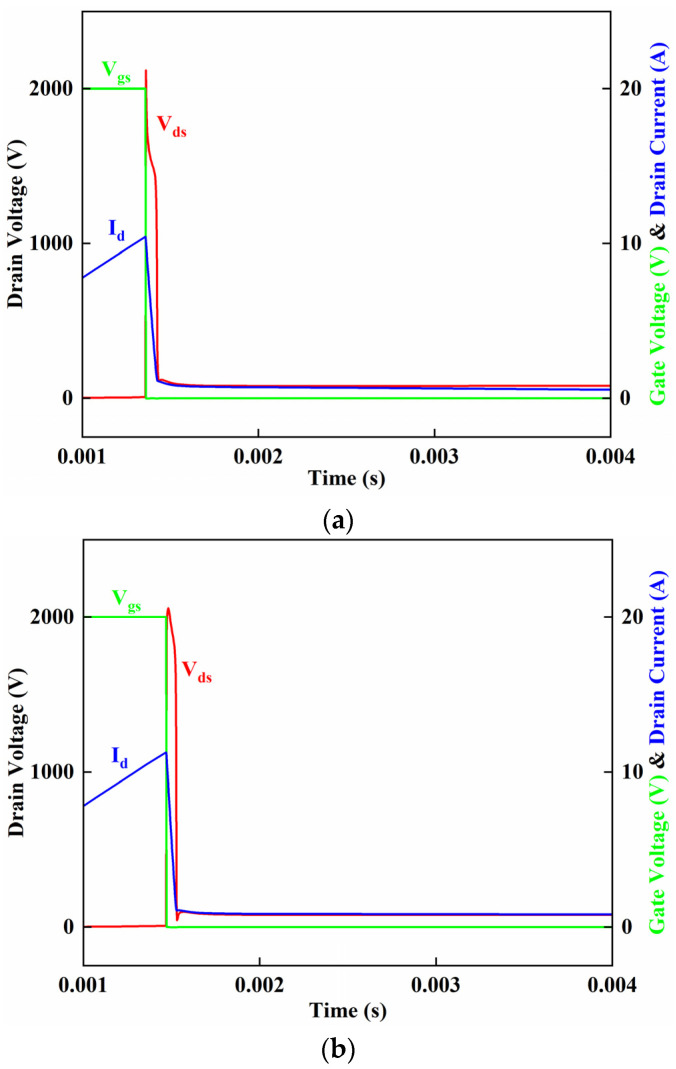
(**a**) The simulated UIS waveform of B-VDMOSFET before failure. (**b**) The simulated UIS waveform of DPNB-VDMOSFET before failure.

The last waveforms before avalanche failure in the B-VDMOSFET, and DPNB-VDMOSFET before UIS failure, are shown in Figure 4a,b. The single avalanche energy Eav of SiC MOSFET is calculated as follows:

(1) $$E_{av}=\frac{1}{2}\cdot L\cdot I_{av}^{2}\cdot \frac{BV}{BV-V_{ds}}$$

The maximum currents before the failure of the B-VDMOSFET and DPNB-VDMOSFET are, respectively, 10.3 A and 11.3 A. This translates to a 1.2× higher avalanche capability for the new design.

**Figure 5:**
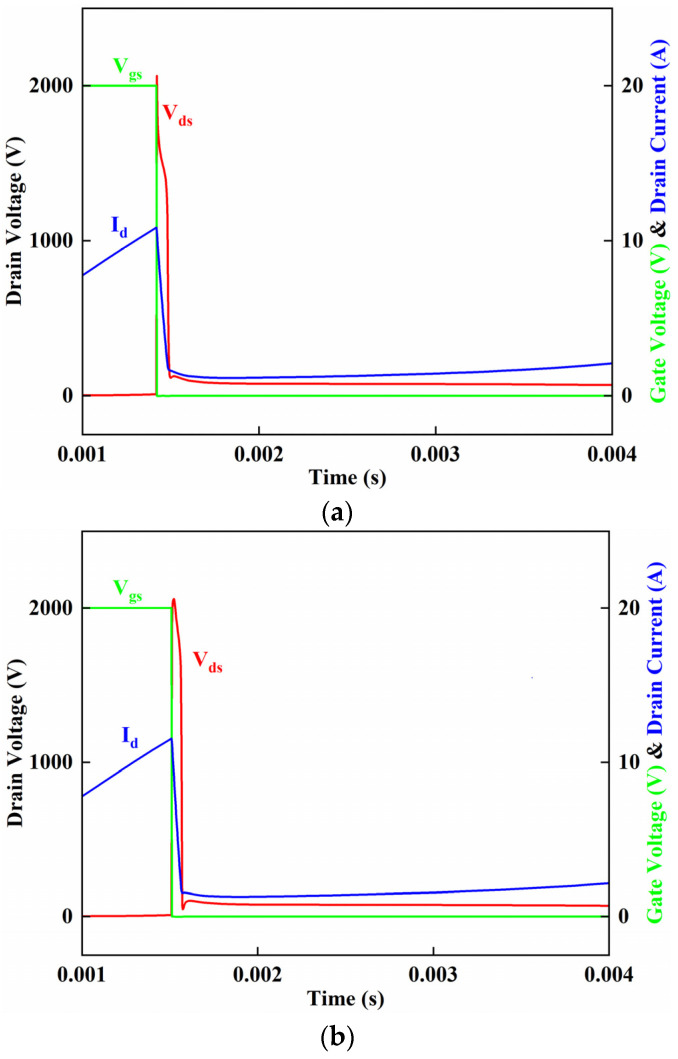
(**a**) The simulated UIS waveform of B-VDMOSFET after failure. (**b**) The simulated UIS waveform of DPNB-VDMOSFET after failure.

The simulated UIS waveforms for B-VDMOSFET and DPNB-VDMOSFET after failure are shown in Figure 5a,b. These waveforms differ slightly from the waveforms measured in [37]. Whether the avalanche current in the device will eventually return to zero is usually used as a criterion for avalanche failure. It can be seen that the currents of the B-VDMOSFET and DPNB-VDMOSFET have a rising trend, so it can be preliminary judged that the B-VDMOSFET and DPNB-VDMOSFET have failed.

It is well known that the avalanche capability of power of SiC MOSFET is closely related to the suppression of parasitic BJT. In order to observe and study the internal physical characteristics of the B-VDMOSFET and DPNB-VDMOSFET, the UIS characteristics of the B-VDMOSFET and DPNB-VDMOSFET were simulated and analyzed by using a 2D computer-aided design (TCAD) physical simulation, and the relevant results were extracted.

**Figure 6:**
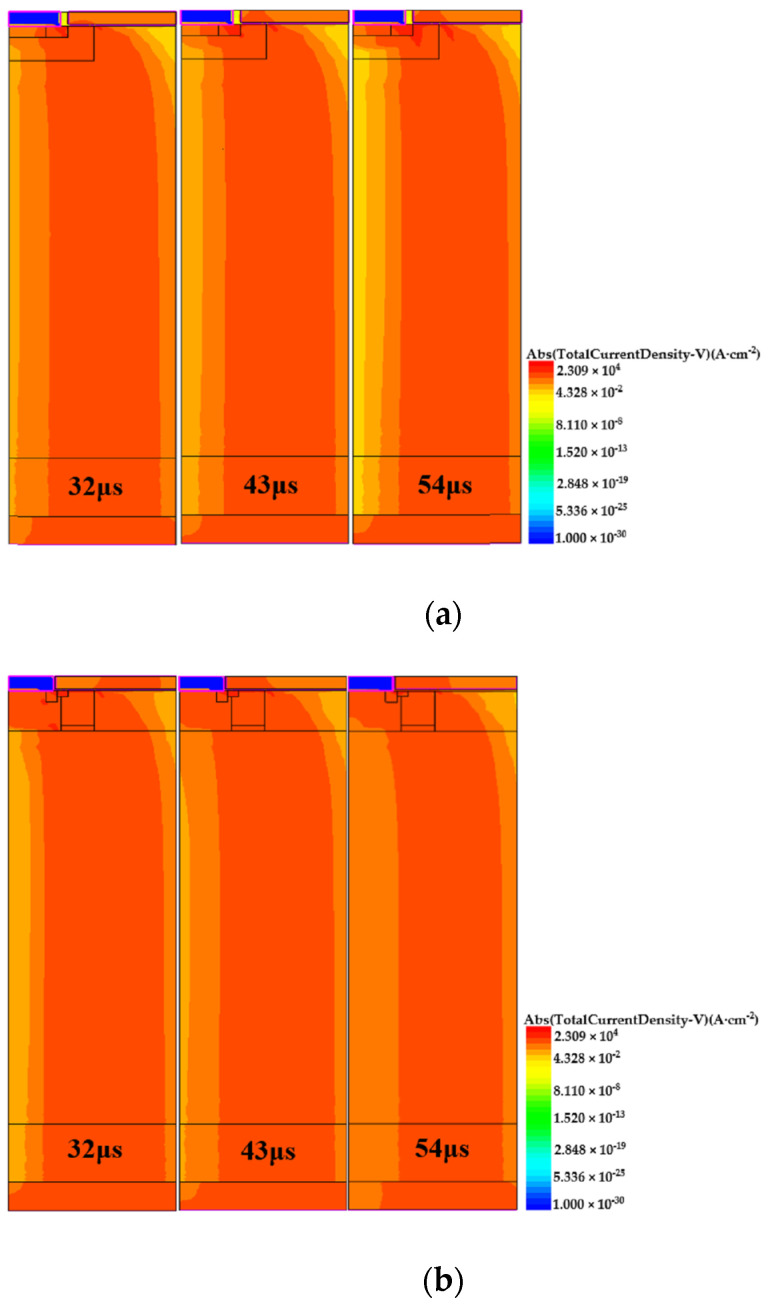
(**a**) The avalanche current density of B-VDMOSFET after failure. (**b**) The avalanche current density of DPNB-VDMOSFET after failure.

Avalanche current density is often used to evaluate the intensity of avalanche breakdown. In order to study the reason for the failure, the distribution of the avalanche current inside B-VDMOSFET and DPNB-VDMOSFET after avalanche failure is extracted by the two-dimensional simulation software Sentaurus TCAD. The avalanche current density of B-VDMOSFET and DPNB-VDMOSFET after avalanche failure is shown in Figure 6a,b. The time shown in the figures is calculated from the discharging phase and the times are taken from beginning of the avalanching.

The parasitic BJT of N-channel SiC MOSFET is NPN-type. When the base-emitter bias voltage is higher than the base-emitter junction built-in voltage, the NPN-type parasitic BJT turns on.

As shown in the figure, there is a large amount of current flowing out of the channel when the B-VDMOSFET and DPNB-VDMOSFET fail in the avalanche. Therefore, we can preliminarily judge that the parasitic BJT is turned on at this time. At the same time, we can see that there is still a large amount of current flowing out of the channel after a period of time, so it can be considered that the parasitic BJT is not closed at this time.

In addition, in Figure 6a,b, the maximum avalanche current density of DPNB-VDMOSFET is much smaller than that of B-VDMOSFET. In Figure 6a, the avalanche current in this B-VDMOSFET is mainly concentrated in the parasitic BJT region. However, as shown in Figure 6b, the avalanche current in DPNB-VDMOSFET is mainly concentrated below the deep P+ region and in the channel at the beginning. This may be one of the reasons why DPNB-VDMOSFET performs better than B-VDMOSFET under UIS conditions.

BJT is a bipolar semiconductor device that generates leakage current even in the reverse-bias PN junction. The thermal generation current generated by SRH recombination and the diffusion current formed by carrier differential concentration constitute the leakage current [38]. The leakage current can be ignored at the low temperature. But, if the temperature gradually increases to be close to the intrinsic temperature of SiC, the leakage current will become non-negligible. 

**Figure 7:**
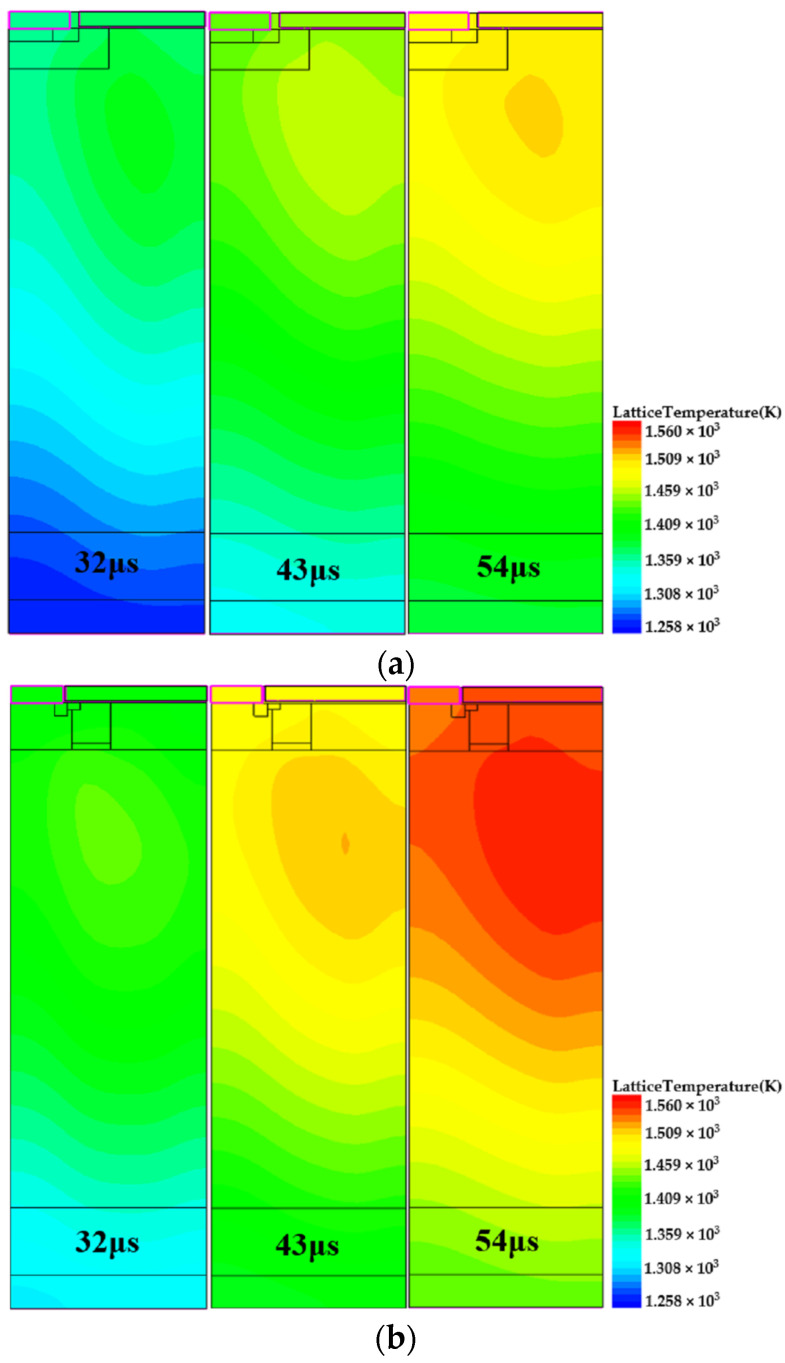
(**a**) The lattice temperature of B-VDMOSFET after failure. (**b**) The lattice temperature of DPNB-VDMOSFET after failure.

To further analyze the cause of avalanche failure in the device, the lattice temperature of B-VDMOSFET and DPNB-VDMOSFET in avalanche mode are shown in Figure 7a,b.The time shown in the figures is calculated from the discharging phase. It can clearly be seen that, after avalanche failure, the B-VDMOSFET first heats from the corner of the P-base region, which gradually diffuses to the entire cell. And in DPNB-VDMOSFET, the temperature changes similarly. The maximum temperatures of B-VDMOSFET and DPNB-VDMOSFET are around 1500 K, and the device may be burned if the temperature inside the device continues to rise.

However, at the time of BJT conduction, the temperature of the channel position has exceeded 1300 K. Such a high temperature will increase the leakage current, which will make it non-ignorable. On the basis of the distribution of electron and hole currents, the combined action of electrons and holes produces the total current through the channel, which is characteristic of the bipolar drain current. Therefore, because the high temperature increases the bipolar leakage current, there is current flowing through the channel even in the absence of a base-emitter positive bias voltage.

Meanwhile, because the built-in voltage of the SiC PN junction has a negative temperature coefficient (NTC), it will gradually decrease when the temperature increases. Such a high temperature, as in Figure 7a,b, causes the junction barrier at the given channel location to drop below 0 V. In the meantime, at high temperatures, the P-base region resistance cannot be ignored, which will result in a positive bias voltage between the parasitic BJT base and emitter, which accelerates the increase in the current of parasitic BJT due to the current amplification effect of bipolar transistor. 

It can be seen from Figure 6a that the current flowing through the P-base region is large when the B-VDMOSFET fails in avalanche. However, in Figure 6b, the current in DPNB-VDMOSFET that flows through P-base region is smaller. This may also be one of the reasons why the DPNB-VDMOSFET has better UIS reliability.

**Figure 8:**
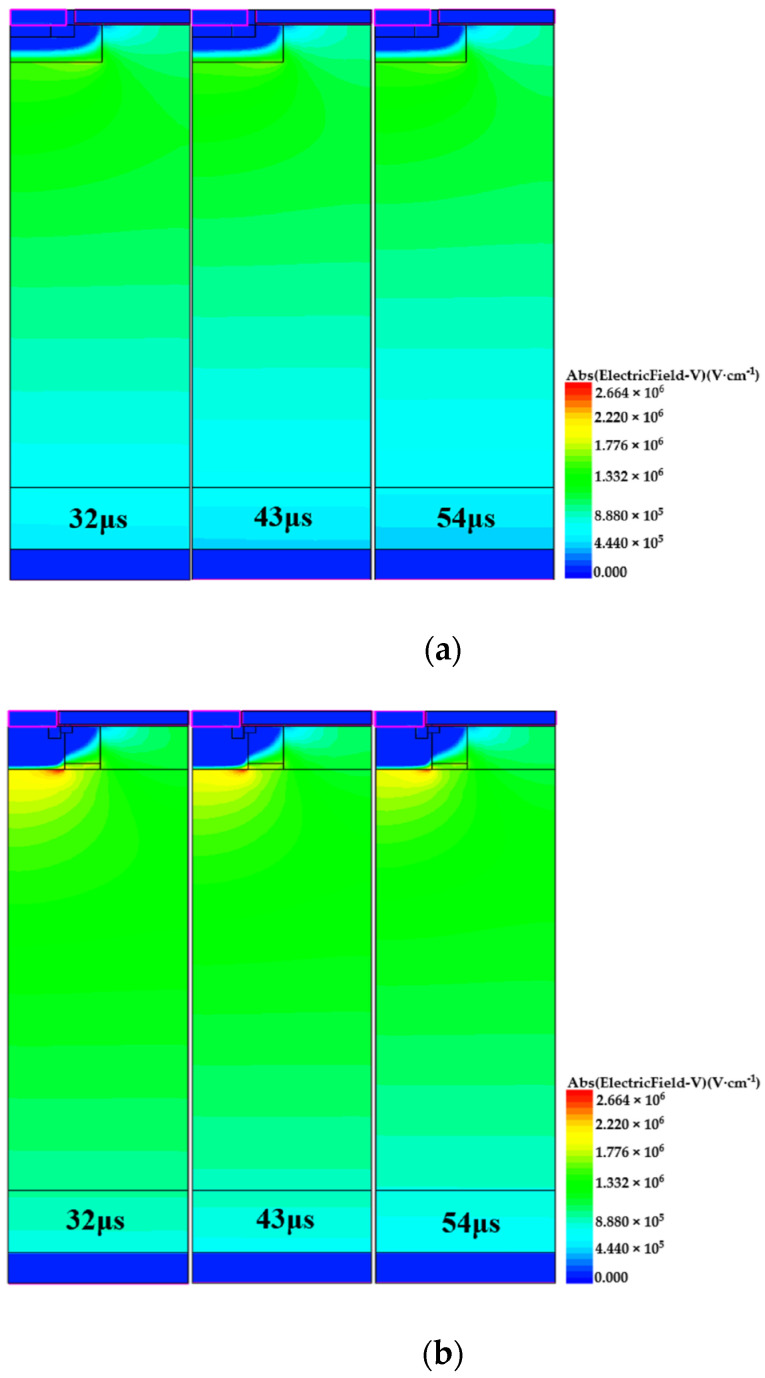
(**a**) The electric field of B-VDMOSFET after failure. (**b**) The electric field of DPNB-VDMOSFET after failure.

In addition, the electric field distribution of the B-VDMOSFET and DPNB-VDMOSFET after failure was extracted using TCAD simulation, as shown in Figure 8a,b. The time shown in the figures is calculated from the discharging phase. As shown in the figure, the highest electric field of B-VDMOSFET is in the corner of the P-base region, while the highest electric field of DPNB-VDMOSFET is in the deep P+ region. At this time, both the electric fields in the gate oxide are less than their highest electric fields. The electric field of B-VDMOSFET does not exceed the electric field limit of SiC material of 3 MV/cm. Due to the presence of the N- region, the electric field of DPNB-VDMOSFET does not reach the breakdown value. The reason that the electric field is high is because there is a lot of current flowing through and a lot of carriers are converging here. 

**Figure 9:**
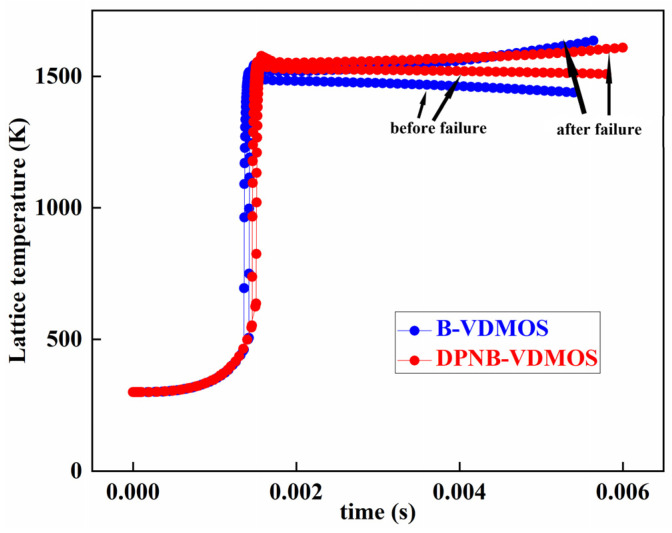
The lattice temperature of B-VDMOSFET and DPNB-VDMOSFET before and after failure.

Figure 9 shows the temperature curve before and after failure of the B-VDMOSFET and DPNB-VDMOSFET. The results in the figure show that the temperature before the failure showed a downward trend, while the temperature of the B-VDMOSFET and DPNB-VDMOSFET both showed a rising trend after the failure. The continuous rise in temperature will eventually lead to thermal runaway of the device.

In conclusion, the reason for the failure of the device may be that the parasitic BJT is on and cannot be turned off, thus it is continuously generating a large amount of current, and further, that the temperature of the device continues to rise until it finally burns out. Therefore, DPNB-VDMOSFET that can inhibit parasitic BJT conduction has better UIS reliability compared to B-VDMOSFET.

## 5. Conclusions

Table 2 shows correlational research about UIS of power devices in the past three years. In this work, DPNB-VDMOSFET improved the avalanche ability and reduced on-resistance.

In this article, the UIS performance of the 1.7-kV VDMOSFET with the same buffer layers was comparatively studied. The device structure proposed under UIS conditions is validated by numerical simulation. The simulation results show that, when L = 10 mH, R = 20 Ω and V_ds_ = 80 V, the avalanche energy of the proposed structure can be increased by 20% compared with the traditional structure. In the meantime, due to adding the CSL layer, the R_on,sp_ of the improved structure is reduced to 1.0 mΩ·cm^2^ compared with another structure, which can reduce the energy lost from the system. 

## Figures and Tables

**Table 1:** Main Device Parameters in Simulations.

Structure Parameters	B-VDMOSFET	DPNB-VDMOSFET
Cell pitch	9.0	9.0
P-base doping concentration (cm^−3^)	2.0 × 10^17^	2.0 × 10^17^
Drift doping concentration (cm^−3^)	3.0 × 10^15^	3.0 × 10^15^
Buffer layer doping concentration (cm^−3^)	5.0 × 10^15^	5.0 × 10^15^
CSL doping concentration (cm^−3^)	–	6.0 × 10^15^
Deep P+ doping Concentration (cm^−3^)	–	1.0 × 10^18^
N− region near N+ doping Concentration (cm^−3^)	–	1.0 × 10^17^
N+ region Concentration (cm^−3^)	1.0 × 10^19^	1.0 × 10^19^
P+ region Concentration (cm^−3^)	1.0 × 10^19^	–
N− region below P-base doping Concentration (cm^−3^)	–	1.0 × 10^15^
Gate oxide Thickness (μm)	0.05	0.05
N+ substrate region Concentration (cm^−3^)	1.0 × 10^19^	1.0 × 10^19^

**Table 2:** Correlational research about UIS of power devices in the past three years.

Structure Name	The Improvement in Avalanche Energy	The Reduction in R_on,sp_ (mΩ·cm^2^)	BV (V)
SiC MOSFETs with varied JFET region width [39]	6.3%	0.5	1200
Asymmetric Trench SiC MOSFET [18]	13.8%	0.1	1200
SiC planar MOSFET [40]	13.3%	-	900
This work	20%	1.0	1700

## Data Availability

The data presented in this study are available on request from the corresponding author. The data are not publicly available due to privacy.

## References

[1] Li Z., Li Z., Zuo C., Fang X. (2022). Application of Nanostructured TiO_2_ in UV Photodetectors: A Review. Adv. Mater..

[2] Li Z., Yan T., Fang X. (2023). Low-dimensional wide-bandgap semiconductors for UV photodetectors. Nat. Rev. Mater..

[3] Li Z., Hong E., Zhang X., Deng M., Fang X. (2022). Perovskite-Type 2D Materials for High-Performance Photodetectors. J. Phys. Chem. Lett..

[4] Parashar S., Kumar A., Bhattacharya S. High Power Medium Voltage Converters Enabled by High Voltage SiC Power Devices. Proceedings of the 2018 International Power Electronics Conference (IPEC-Niigata 2018-ECCE Asia).

[5] Bhatnagar M., Baliga B.J. (1993). Comparison of 6H-SiC, 3C-SiC, and Si for power devices. IEEE Trans. Electron Devices.

[6] Adappa R., Suryanarayana K., Hatwar H.S., Rao M.R. Review of SiC based Power Semiconductor Devices and their Applications. Proceedings of the 2019 2nd International Conference on Intelligent Computing, Instrumentation and Control Technologies (ICICICT).

[7] Williams R.K., Darwish M.N., Blanchard R.A., Siemieniec R., Rutter P., Kawaguchi Y. (2017). The Trench Power MOSFET—Part II: Application Specific VDMOS, LDMOS, Packaging, and Reliability. IEEE Trans. Electron Devices.

[8] Kelley M.D., Pushpakaran B.N., Bayne S.B. (2017). Single-Pulse Avalanche Mode Robustness of Commercial 1200 V/80 mΩ SiC MOSFETs. IEEE Trans. Power Electron..

[9] Ren N., Wang K.L., Wu J., Xu H., Sheng K. Failure Mechanism Analysis of SiC MOSFETs in Unclamped Inductive Switching Conditions. Proceedings of the 2019 31st International Symposium on Power Semiconductor Devices and ICs (ISPSD).

[10] Fayyaz A., Castellazzi A., Romano G., Riccio M., Irace A., Urresti J., Wright N. UIS failure mechanism of SiC power MOSFETs. Proceedings of the 2016 IEEE 4th Workshop on Wide Bandgap Power Devices and Applications (WiPDA).

[11] Ren N., Hu H., Wang K.L., Zuo Z., Li R., Sheng K. Investigation on single pulse avalanche failure of 900V SiC MOSFETs. Proceedings of the 2018 IEEE 30th International Symposium on Power Semiconductor Devices and ICs (ISPSD).

[12] Nida S., Kakarla B., Ziemann T., Grossner U. (2021). Analysis of Current Capability of SiC Power MOSFETs under Avalanche Conditions. IEEE Trans. Electron Devices.

[13] Zhao X., Li T., Liang X., Wu Y., Li C., Zheng Z. Investigation on SiC MOSFET’s Avalanche and Short-Circuit Failure Mechanism. Proceedings of the 2022 IEEE 5th International Electrical and Energy Conference (CIEEC).

[14] ACastellazzi, Fayyaz A., Romano G., Riccio M., Irace A., Urresti J., Wright N. Transient out-of-SOA robustness of SiC power MOSFETs. Proceedings of the 2017 IEEE International Reliability Physics Symposium (IRPS).

[15] Bai Z., Tang X., Xie S., He Y., Yuan H., Song Q., Zhang Y. (2021). Investigation on Single Pulse Avalanche Failure of 1200-V SiC MOSFETs via Optimized Thermoelectric Simulation. IEEE Trans. Electron Devices.

[16] Qi J., Yang X., Li X., Chen W., Long T., Tian K., Hou X., Wang X. (2021). Comprehensive Assessment of Avalanche Operating Boundary of SiC Planar/Trench MOSFET in Cryogenic Applications. IEEE Trans. Power Electron..

[17] An J., Hu S. (2020). Experimental and Theoretical Demonstration of Temperature Limitation for 4H-SiC MOSFET During Unclamped Inductive Switching. IEEE J. Emerg. Sel. Top. Power Electron..

[18] Deng X., Zhu H., Li X., Tong X., Gao S., Wen Y., Bai S., Chen W., Zhou K., Zhang B. (2020). Investigation and Failure Mode of Asymmetric and Double Trench SiC mosfets Under Avalanche Conditions. IEEE Trans. Power Electron..

[19] Li X., Tong X., Hu R., Wen Y., Deng X., Sun Y., Chen W., Ba S., Zhang B. (2021). Failure Mechanism of Avalanche Condition for 1200-V Double Trench SiC MOSFET. IEEE J. Emerg. Sel. Top. Power Electron..

[20] Yao K., Yano H., Iwamuro N. (2022). Impact of Negative Gate Bias and Inductive Load on the Single-Pulse Avalanche Capability of 1200-V SiC Trench MOSFETs. IEEE Trans. Electron Devices.

[21] Yao K., Yano H., Iwamuro N. Investigations of UIS failure mechanism in 1.2 kV trench SiC MOSFETs using electro-thermal-mechanical stress analysis. Proceedings of the 33rd International Symposium on Power Semiconductor Devices and ICs (ISPSD).

[22] Wei J., Liu S., Li S., Fang J., Li T., Sun W. (2019). Comprehensive Investigations on Degradations of Dynamic Characteristics for SiC Power MOSFETs Under Repetitive Avalanche Shocks. IEEE Trans. Power Electron..

[23] Mao H., Qiu G., Jiang X., Jiang H., Zhong X., Tang L., Zhang Y., Ran L., Wu Y. (2022). Investigation on the Degradations of Parallel-Connected 4H-SiC MOSFETs under Repetitive UIS Stresses. IEEE Trans. Electron Devices.

[24] Zhou X., Su H., Yue R., Dai G., Li J., Wang Y., Yu Z. (2018). A Deep Insight Into the Degradation of 1.2-kV 4H-SiC mosfets under Repetitive Unclamped Inductive Switching Stresses. IEEE Trans. Power Electron..

[25] Liu S., Gu C., Wei J., Qian Q., Sun W., Huang A.Q. (2016). Repetitive Unclamped-Inductive-Switching-Induced Electrical Parameters Degradations and Simulation Optimizations for 4H-SiC MOSFETs. IEEE Trans. Electron Devices.

[26] Deng X., Huang W., Li X., Li X., Chen C., Wen Y., Ding J., Chen W., Sun Y., Zhang B. (2022). Investigation of Failure Mechanisms of 1200 V Rated Trench SiC MOSFETs under Repetitive Avalanche Stress. IEEE Trans. Power Electron..

[27] Marek J., Minárik M., Matúš M., Kozárik J., Stuchlíková L. SiC Power TrenchMOS Transistor under harsh repetitive switching conditions. Proceedings of the 2023 25th European Conference on Power Electronics and Applications (EPE’23 ECCE Europe).

[28] Marek J., Kozárik J., Minárik M., Chvála A., Matúš M., Stuchlíková L. SiC Power TrenchMOS Transistor Under Repetitive Avalanche Stress. Proceedings of the 2022 14th International Conference on Advanced Semiconductor Devices and Microsystems (ASDAM).

[29] Zhu Z., Ren N., Xu H., Liu L., Sheng K. (2021). Characterization and Analysis on Performance and Avalanche Reliability of SiC MOSFETs with Varied JFET Region Width. IEEE Trans. Electron Devices.

[30] Liu S., Titus J.L., Boden M. (2007). Effect of Buffer Layer on Single-Event Burnout of Power DMOSFETs. IEEE Trans. Nucl. Sci..

[31] Luo J.-H., Wang Y., Bao M.-T., Li X.-J., Yang J.-Q., Cao F. (2022). Simulation Study of Single-Event Burnout Reliability for 1.7-kV 4H-SiC VDMOSFET. IEEE Trans. Device Mater. Reliab..

[32] Goh J., Kim K. High Efficiency 1700V 4H-SiC UMOSFET with Local Floating Superjunction. Proceedings of the 2020 International Conference on Electronics, Information, and Communication (ICEIC).

[33] Titus J.L., Wheatley C.F., Van Tyne K.M., Krieg J.F., Burton D.I., Campbell A.B. (1998). Effect of ion energy upon dielectric breakdown of the capacitor response in vertical power MOSFETs. IEEE Trans. Nucl. Sci..

[34] Abbate C., Busatto G., Tedesco D., Sanseverino A., Velardi F., Wyss J. (2019). Gate Damages Induced in SiC Power MOSFETs During Heavy-Ion Irradiation—Part II. IEEE Trans. Electron Devices.

[35] (2018). ATLAS User’s Manual: Device Simulation Software.

[36] Zhou J.-C., Wang Y., Li X.-J., Yang J.-Q., Bao M.-T., Cao F. (2022). Simulation Study of Single-Event Effects for the 4H-SiC VDMOSFET With Ultralow On-Resistance. IEEE Trans. Electron Devices.

[37] Chen H., Wang J., Dong R., Yao J., Liang S. Investigation on Single Pulse Avalanche Failure of 3.3kV planar-SiC power MOSFETs. Proceedings of the 2023 IEEE PELS Students and Young Professionals Symposium (SYPS).

[38] Wang Z., Shi X., Tolbet L.M., Wang F., Liang Z., Costinett D., Blalock B.J. (2016). Temperature-Dependent Short-Circuit Capability of Silicon Carbide Power MOSFETs. IEEE Trans. Power Electron..

[39] Zhu Z., Ren N., Xu H., Liu L., Sheng K. Avalanche Reliability of Planar-gate SiC MOSFET with Varied JFET Region Width and Its Balance with Characteristic Performance. Proceedings of the 2021 33rd International Symposium on Power Semiconductor Devices and ICs (ISPSD).

[40] Gao Z., Cao L., Guo Q., Sheng K. Experimental Investigation of the Single Pulse Avalanche Ruggedness of SiC Power MOSFETs. Proceedings of the 2020 IEEE Applied Power Electronics Conference and Exposition (APEC).

